# Potential Role of Lymphovenous Bypass in Mitigating Alzheimer's Disease Dementia

**DOI:** 10.1055/a-2627-9243

**Published:** 2025-07-23

**Authors:** Qingping Xie, Changsik John Pak, Jingeun Kwon, Sung-Chuan Chao, Joon Pio Hong

**Affiliations:** 1 Department of Plastic and Reconstructive Surgery, Hangzhou Qiushi Hospital, Hangzhou, Zhejiang, People's Republic of China; 2 Department of Plastic and Reconstructive Surgery, University of Ulsan, College of Medicine, Seoul Asan Medical Center, Seoul, Korea (the Republic of); 3 Department of Traumatology, National Taiwan University Hospital, National Taiwan University College of Medicine, Taipei, Taiwan

**Keywords:** Alzheimer's disease, dementia, behavioral and psychological symptoms, brain lymphatics, lymphovenous bypass

## Abstract

This case report explores the therapeutic potential of lymphovenous bypass (LVB) surgery performed at the neck in neurodegenerative diseases, specifically Alzheimer's disease (AD) dementia. The subject is a 58-year-old woman who was previously healthy but began experiencing unexplained memory decline and frequent disorientation in the past 7 years, leading to an AD diagnosis. Despite ongoing pharmaceutical therapy, her symptoms progressed to severe dementia accompanied by behavioral and psychological symptoms of dementia (BPSD). Her Mini-Mental State Examination (MMSE) and Montreal Cognitive Assessment (MoCA) scores were both 0/30, and ^18^ F-AV-45 PET/CT revealed abnormal brain amyloid load. For salvage therapy, she underwent LVBs on her neck bilaterally. Postoperatively, she got better MMSE and MoCA scores with dramatic improvement in communication and activity. ^18^ F-AV-45 PET/CT scans 4 months after surgery indicated a reduction in abnormal brain amyloid deposits. This case report highlights the potential effectiveness of LVB surgery in reducing brain amyloid load and attenuating cognitive impairment and BPSD. Further research with animal experiments and clinical trials is necessary to confirm these findings.

## Introduction

Exploring the anatomical pathways responsible for cerebrospinal fluid (CSF) and interstitial fluid circulation in humans—including the glymphatic (glial–lymphatic) and meningeal routes—highlights one of the key exit points for cranial CSF that eventually directs its flow into the cervical lymphatic system. 1 2 3 Moreover, impairment of the meningeal lymphatic system may worsen the progression of Alzheimer's disease (AD) and play a role in the cognitive decline associated with aging. 4 Recently, we have recognized the pathophysiological significance of dementia in individuals diagnosed with head and neck cancer. Those who undergo bilateral and comprehensive lymph node dissection exhibit an increased risk of developing dementia. 5 Conversely, the neck lymphatics, potentially enriched with amyloid-β (Aβ) deposits, could serve as a theoretically accessible site to enhance glymphatic drainage and help mitigate dementia. 6 Hence, we hypothesize that bypassing the brain lymphatics from a degenerated lymphatic system into a larger-caliber and faster-drainage venous system at the neck will accelerate brain metabolic waste clearance. The proposal of utilizing extracranial lymphatic reconstruction to “declog” the brain and improve brain health has been made, with scarce articles published on the topic. 7 8 Therefore, we are keen to explore the therapeutic potential of lymphovenous bypass (LVB) performed at the neck in neurodegenerative diseases, specifically AD, with evidence from cognitive assessments, positron emission tomography/computed tomography, and video clip demonstrations over the last 4-month follow-up period.

## Case

A 58-year-old female patient was in good health before experiencing unexplained memory decline and frequent disorientation 7 years ago. She did not seek medical aid initially, and her condition progressively worsened, marked by short-term memory loss and spatial disorientation. After visiting two medical centers in Beijing and Shanghai, China, and she was diagnosed with AD when she was 53 years old. The patient had been receiving conventional AD medications for several years, including the cholinesterase inhibitor donepezil (10 mg once daily in the evening) and the N-methyl-D-aspartate (NMDA) receptor antagonist memantine (10 mg twice daily). Despite pharmaceutical treatment, her symptoms progressively worsened, leading to hallucinations, speech disorders, depression, apathy, and walking difficulties due to muscle spasticity. To address her psychiatric symptoms, sertraline, a selective serotonin reuptake inhibitor, was prescribed as an antidepressant, while risperidone (1 mg) was occasionally administered to manage hallucination episodes. As previously mentioned, her clinical presentation was consistent with severe AD dementia with behavioral and psychological symptoms.

**Fig. 1:**
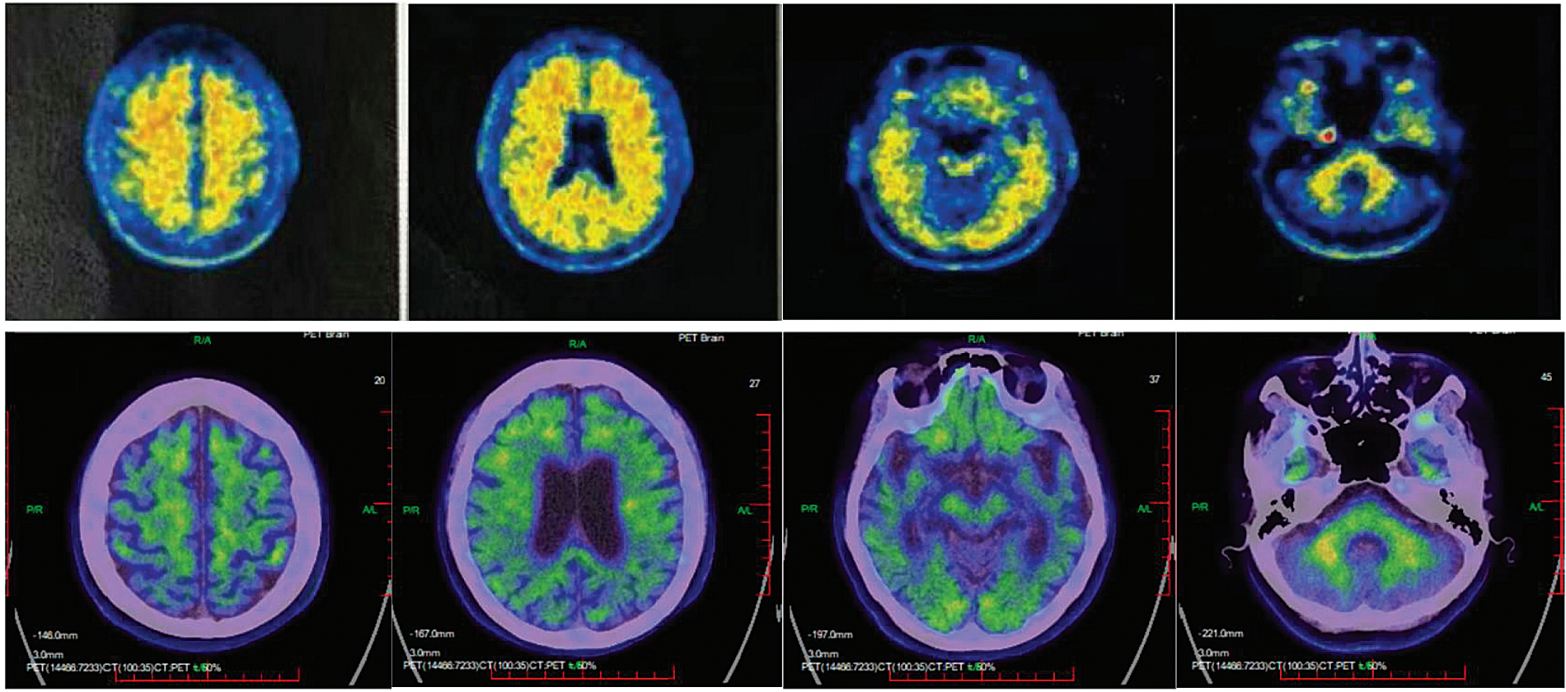
The ^18^ F-AV-45 PET/CT, a medical imaging technique used to detect amyloid plaques in the brain, a hallmark of Alzheimer's disease, confirmed an abnormal brain amyloid load. Preoperative 4-year scan (upper row) and postoperative 4-month scan (lower row).

The Mini-Mental State Examination (MMSE) and the Montreal Cognitive Assessment (MoCA) are tools designed for systematic and thorough assessment of mental status. The MoCA, in particular, has been validated as a highly sensitive tool for the early detection of mild cognitive impairment (MCI). Upon presentation, her MMSE and MoCA scores were both 0/30. The 18F-AV-45 PET/CT scan, a medical imaging technique used to detect amyloid plaques in the brain, is a hallmark of AD. It involves injecting a radioactive tracer (Florbetapir F-18) that binds to amyloid deposits, allowing doctors to visualize them on PET/CT scans, aiding early diagnosis and treatment planning. It showed standardized uptake value ratio (SUVr) of 1.53 in the frontal cortex, 1.57 in the parietal cortex, 1.50 in the temporal cortex, and 1.60 in the posterior cingulate. Additionally, the centiloid (CL) scale measured approximately 100 CL, suggesting at least mild-to-moderate AD and indicating advanced amyloid/tau accumulation in the brain in specific regions of interest ( Fig. 1 and Table 1 ). 9 10 11 12 With a full understanding of her condition and the limited treatment options available, her family decided to proceed with pilot LVB surgery, providing informed consent for both the procedure and the use of her photos, videos, and medical records.

**Table 1:** The ^18^ F-AV-45 (florbetapir) PET/CT value/ratio and centiloid before and after surgery

Regions of interest SUV value/ratio and CL		Frontal cortex	Parietal cortex	Temporal cortex	Posterior cingulate	Cerebellum
Preoperative	SUV	1.07	1.10	1.05	1.12	0.7
	SUVr	1.53	1.57	1.5	1.6	–
	CL	102.8	110.2	97.3	115.7	–
Postoperative 4 months	SUV	1.288	1.145	1.045	1.27	1.016
	SUVr	1.27	1.13	1.03	1.25	–
	CL	55.2	29.6	11.3	51.6	–

Abbreviations: CL, centiloid; SUV, standardized uptake value; SUVr, standardized uptake value ratio, the regional-to-cerebellum comparison.

### Surgical Approaches

**Fig. 2:**
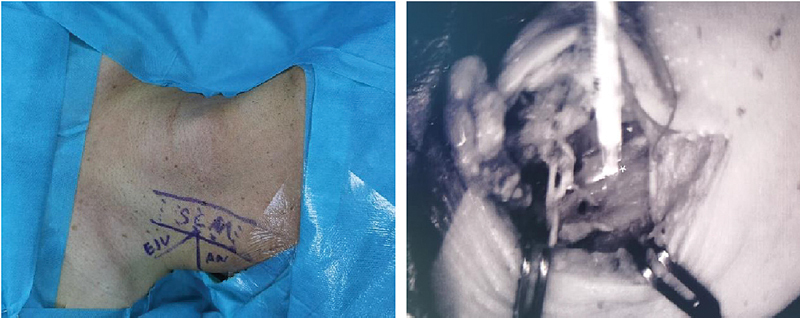
Using ultrasound mapping, we preoperatively marked the sternocleidomastoid (SCM) muscle, external jugular vein (EJV), accessory nerve (AN), and lymph nodes (left side). Indocyanine green (ICG) was injected cranially along the carotid sheath (white star) to trace the lymphatic channels and confirm their patency following lymphovenous bypass.

**Fig. 3:**
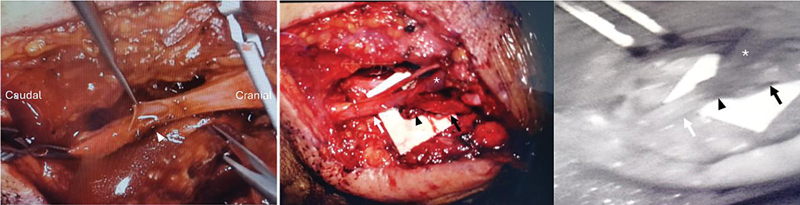
The lymphovenous bypass at the neck was performed by making a longitudinal incision on the external jugular vein (EJV; white arrowhead; left). Lymphatic channels, confirmed with Indocyanine green (ICG) enhancement, were connected to the EJV under a microscope by “octopus” technique (middle). Anastomosis (black arrowhead) was accomplished with a patency check (right). Lymphatic channels (black arrow) and the EJV distal to the anastomosis (white arrow) showed ICG signal, while the cranial EJV was not enhanced (white star).

1. Positioning and Marking: Under general anesthesia, the patient is placed in a supine position with the head rotated to expose the surgical area. Marks are made at the posterior edge of the sternocleidomastoid muscle and the external jugular vein (EJV) for precise localization.

1. Incision Design and Fluorescence Tracing: A 6-cm horizontal incision is made at the middle point of the sternocleidomastoid muscle posterior edge to fully expose the deep cervical lymphatic system 12 13 and the EJV. The accessory nerve should be carefully preserved. Diluted (1:100) indocyanine green (ICG) fluorescent dye is injected along the carotid sheath cranially to approach the jugular foramen as closely as possible using an 18-gauge blunt needle ( Fig. 2 ). 12 The lymphatic flow pathway is then illustrated along the carotid sheath, ensuring the lymphatic vessels can be clearly observed under the microscope with a near-infrared wavelength camera.

1. Microsurgical Anastomosis Technique: Using the “octopus” technique, multiple enlarged lymphatic vessels are connected to the EJV in an end-to-side fashion. 14 When the lymphatic channels are too small, lymph node-to-vein anastomosis (LNVA), by connecting the branches of the EJV to a perforated opening in the neck lymph node. This can be included as part of the protocol to divert brain lymphatics. 15 All anastomoses are ensured to achieve optimal fluid flow without blockages or leakage under a microscope ( Fig. 3 ).

1. After meticulous hemostasis and saline irrigation, the neck wounds were closed in layers with the insertion of a closed system drain tube. The same procedure was performed on the contralateral neck.

### Postoperative Course and Rehabilitation

Typically, the surgery takes under 3 hours, supported by thorough preoperative preparation and accurate intraoperative lymphatic exploration. The brief duration of general anesthesia minimizes the risk of postoperative delirium, which, if it occurs, generally subsides within 1 to 2 days. Preserving prior medications throughout the perioperative period and ensuring standard ward admission with consideration for circadian rhythm can further help prevent delirium.

Patients routinely require 1 week of in-hospital observation, followed by a 2-week comprehensive rehabilitation program that primarily includes language, walking, and deformity correction training. For our patient, her postoperative course was uneventful, and subsequent cognitive tests were assessed.

### Results

**Video 1** Demonstration of the index patient before and after lymphovenous bypass surgery.


During the early postoperative period, the clinicians were maintaining her prior medications for AD dementia and behavioral and psychological symptoms of dementia (BPSD). To carefully mitigate the learning effect, the patient's MMSE and MoCA scores increased from 0 to 4 and 0 to 2, respectively, at 1-month postsurgery compared with the preoperative evaluation, with notable advance in “Registration” and “Language” on the MMSE and “Naming” on the MoCA, as shown in Table 2 . Although the scores still indicated severe impairment, the video clip showed significant improvement in her communication, engagement in activities, mood stability, and motility due to relief from muscle spasticity ( Video 1 ). Her progress was sustained for 4 months after the surgical intervention. At 4 months postoperatively, the ^18^ F-AV-45 PET/CT revealed decreasing SUVr values—1.27 in the frontal cortex, 1.13 in the parietal cortex, 1.03 in the temporal cortex, and 1.25 in the posterior cingulate—along with a decline in CL values, indicating a reduction in abnormal brain amyloid deposition ( Fig. 1 and Table 1 ).

**Table 2:** The score of MMSE and MoCA before and after surgery

Event	MMSE (total 30)	MoCA (total 30)
Preoperative	0	0
Postop day 4	1	1
Postop 1 week	3	1
Postop 1 month	4	2
Postop 4 months	4	2

Abbreviations: MMSE, Mini-Mental State Examination; MoCA, Montreal Cognitive Assessment.

## Discussion

To our knowledge, this case report provides the most detailed account of the clinical course, including diagnosis of AD dementia and accompanying behavioral and psychological symptoms, limited response to standardized medical treatment, cognitive tests, and amyloid brain imaging studies. 16 We also describe the surgical rationale and procedures step-by-step, demonstrating the longest follow-up with promising results in the current literature.

Regarding the indications for LVB surgery to attenuate AD dementia, we only included patients with a definite diagnosis made by neurologists, including cognitive test and imaging evidence, and who had been approved after failed medical therapy and other standard treatments. Based on the hypothesis that brain waste could be expelled from deep cervical lymphatic systems, which may become dysfunctional due to aging or other insults, we developed an innovative shunt between the deep cervical lymphatics and neck veins to enhance waste clearance. As a result, the abnormal brain amyloid load was reduced, leading to an improvement in the accompanying clinical symptoms.

In our case, a severe AD dementia patient showed clinical improvement in the video clip, despite not making significant progress on the MMSE and MoCA. Remarkably, we observed that the cognitive improvement peaked at 1 month postoperatively and then sustained throughout the 4-month follow-up period. Over and above that, we observed significant improvement in her spastic posture after surgery, allowing her to ambulate independently, as shown in Video 1 . Meanwhile, we noticed a dramatic improvement in the patient's communication, engagement in activities, and mood stability. Before surgery, she experienced hallucinations, speech disorders, depression, and apathy—symptoms commonly associated with BPSD, which often have a greater impact on quality of life than cognitive impairment itself. 17 18 This suggests the potential for improvement in various neurodegenerative diseases by enhancing brain waste clearance through this bypass surgery. According to our preliminary results (not shown), the milder the dementia symptoms the patients had before surgery, the better the outcomes. As our experience grows, we aim to identify the most effective indications for neurodegenerative patients to alleviate dementia symptoms through surgical intervention.

We further analyzed the changes in SUVr from ^18^ F-AV-45 PET/CT and found a postsurgical decrease. Given that normal individuals typically exhibit lower SUVr values than those with AD, this trend aligns with the observed reduction in CL scale, suggesting less disease progression. These findings indicate a decrease in brain amyloid load in key regions of interest, providing additional indirect yet imaging-based evidence of the surgery's efficacy. 10 19

**Fig. 4:**
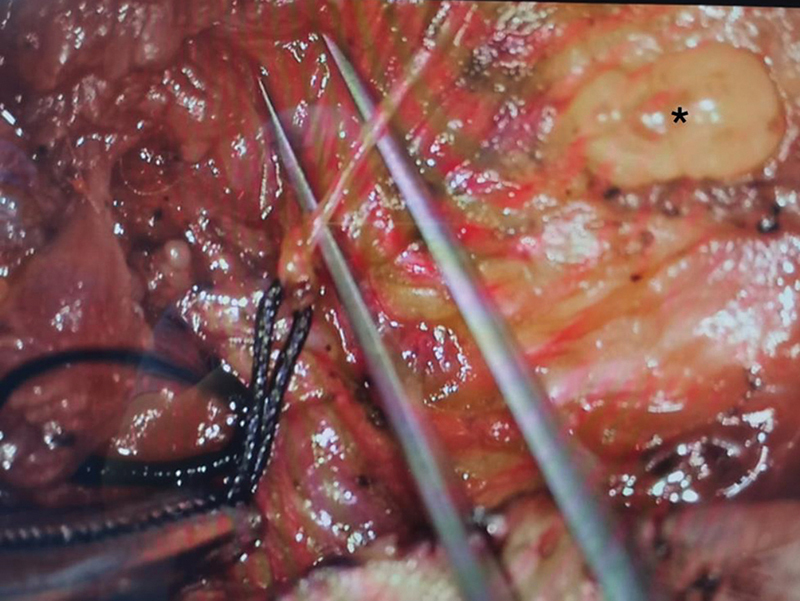
The pathologically enlarged lymph node (black star) and the branch vein of external jugular vein (EJV) were dissected (above forceps) for lymph node-to-vein anastomosis (LNVA).

**Fig. 5:**
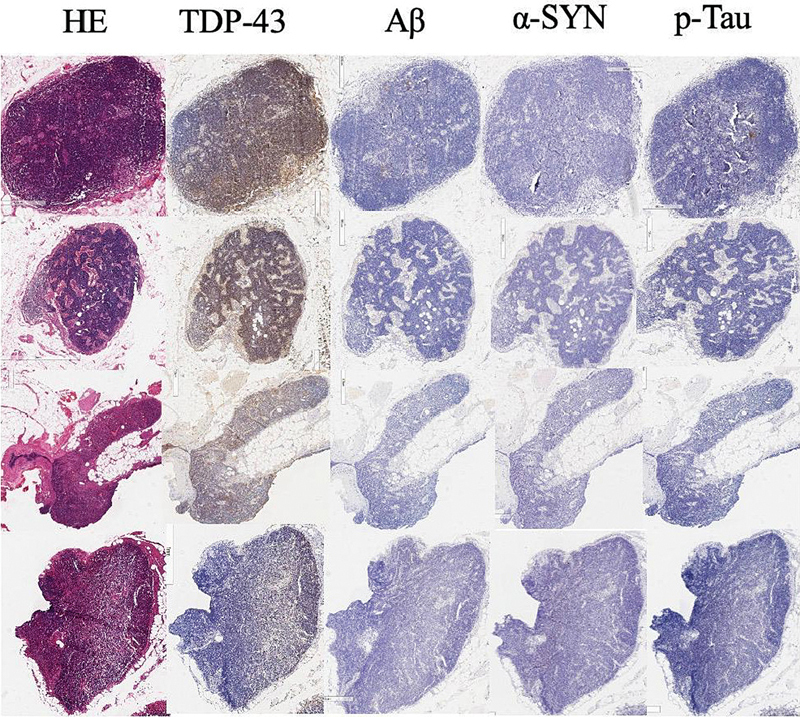
Pathohistological examination of neck lymph nodes showed HE stains, TDP-43, amyloid-β (Aβ), phosphorylated tau (p-tau) (dementia proteins), α-synuclein (pathological factor). TDP-43, transactive response DNA binding protein 43.

The key to successfully performing LVB surgery at the neck is to confirm the lymphatic channels or lymph nodes drained from the brain. Precise and safe ICG injections can identify the targeted lymphatic tissues and help ensure patency after bypass surgery ( Fig. 3 , right). Meanwhile, pathologically enlarged lymph nodes in deep cervical areas make LNVA easier to perform ( Fig. 4 ). Figure 5 illustrates the pathohistological study of cervical lymph nodes sampled during surgery, focusing on specific proteins—TDP-43 (transactive response DNA binding protein 43), Aβ, α-synuclein, and phosphorylated tau (p-tau). These proteins serve as markers for neurodegenerative conditions, including amyotrophic lateral sclerosis, AD, Parkinson's disease, and tauopathies such as Alzheimer's, respectively. It provided pathological evidence of AD diagnosis.

There are several limitations to our report. First, we have only one patient with comprehensive studies and documentation. A larger patient cohort, extended follow-up periods, and official clinical trial registration are warranted. Moreover, we cannot determine exactly which severity of AD dementia or patients with other neurodegenerative diseases will benefit most from surgery. CSF biomarkers for the early stage of AD, such as Aβ42 (one of the two main isoforms of Aβ and a major constituent of Aβ plaques), ratios of these CSF biomarkers (e.g., Aβ42/40), p-tau, and total tau (t-tau), could be used to objectively validate postoperative changes. 20 21 22 Recently, the plasma p-tau 217 immunoassay has been developed to accurately identify biological AD through a blood test. 23 Hence, a well-designed study with rigorous patient selection and the application of objective biomarkers should be considered for future research. Third, surgical procedures should be revisited to obtain the most effective results. Lastly, we were unable to directly measure the clearance of brain waste before and after surgery in clinical scenarios; instead, we relied on indirect assessments based on clinical presentations, cognitive tests, and imaging studies. Experimental animal (rodent) models should be used to verify the effectiveness of LVBs and to uncover the mechanisms in brain health, which demand immediate attention. 24

In summary, this case report highlights the potential effectiveness of LVB surgery in reducing brain amyloid load and attenuating motor and cognitive impairment. Further research with larger patient groups and clinical trials is necessary to confirm these findings.
